# Adolescent mothers: A qualitative study on barriers and facilitators to mental health in a low-resource setting in Cape Town, South Africa

**DOI:** 10.4102/phcfm.v12i1.2279

**Published:** 2020-05-28

**Authors:** Sally Field, Zulfa Abrahams, Simone Honikman

**Affiliations:** 1 Perinatal Mental Health Project, Alan J. Flisher Centre for Public Mental Health, Department of Psychiatry and Mental Health, Faculty of Health Sciences, University of Cape Town, Cape Town, South Africa

**Keywords:** adolescents, maternal mental health, service uptake, barriers to care, facilitators to care, qualitative, depression, anxiety

## Abstract

**Background:**

Pregnant and postnatal adolescent women are a high-risk group for common mental disorders (CMDs); however, they have low levels of engagement and retention with mental health services. Negative consequences of CMDs have been documented for both mother and child.

**Aim:**

The study aimed to explore the barriers and facilitators to service access for adolescents in low-resource settings.

**Setting:**

We interviewed 12 adolescents, aged 15–19 years, from low-resource settings in Cape Town, South Africa. Participants had previously engaged with a mental health service, integrated into maternity care.

**Methods:**

Twelve semi-structured, individual interviews were used for this qualitative study. Interviews were recorded, transcribed and coded. A framework analysis was employed for data analysis.

**Results:**

Adolescents perceived considerable stigma around both teenage pregnancy and mental illness, which inhibited use of mental health services. Other barriers included fearing a lack of confidentiality as well as logistical and environmental obstacles. Service uptake was facilitated by support from other adults and flexible appointment times. Face-to-face individual counselling was their preferred format for a mental health intervention.

**Conclusion:**

Several key components for adolescent-friendly mental health services emerged from our findings: integrate routine mental health screening into existing obstetric services to de-stigmatise mental health problems and optimise screening coverage; coordinate obstetric and counselling appointment times to rationalise the use of limited resources; and sensitise care providers to the needs of adolescents to reduce stigma around adolescent sexual activity and mental illness. A non-judgemental, caring and confidential relationship between counsellors and clients is crucial for successful interactions.

## Introduction

Of the approximate 16 million adolescent girls who give birth annually, 95% of these births occur in low-and middle-income countries (LMICs).^1^ Globally, however, adolescents display low levels of help-seeking behaviour and poor retention in health services.^2^

The adolescent growth and consolidation phase, which occurs between the ages of 15 and 19 years, is a time of brain restructuring, linked to exploration, experimentation and the initiation of behaviours that are lifelong determinants of health.^3^ In addition to negotiating adolescent development, teenage mothers are also faced with the challenges of adapting to new roles and responsibilities of parenting. Pregnant and parenting adolescents have been shown to be at an increased risk for adverse physical and mental health outcomes compared with their adult counterparts.^4^

In LMICs, complications from pregnancy and childbirth are the leading cause of mortality in women aged 15–19 years.^5^ Compared with women aged 20–24 years, younger women are more likely to suffer from anaemia in the perinatal period.^6^ Perinatal deaths are 50% higher among babies born to this age group than among babies born to women aged between 20 and 29 years. Newborns of adolescent mothers are more likely to have lower birth weights.^5^ In LMICs, studies show that low wealth quintile, limited media exposure, rural residence and low education level of both the adolescent and her partner negatively influenced the use of maternal health services.^7^

When compared with the pregnant adult population, pregnant adolescents are at increased risk for common mental disorders (CMDs) such as depression and anxiety.^8^ The risk factors for CMDs during and after pregnancy for adolescents include inadequate social and familial support, socio-economic hardship, prior experience of depression, unintended pregnancy and a negative societal perception of teenage pregnancy.^8,9^ A recent systematic review of studies from developed countries showed that adolescent mothers report extreme stress from conflict with parents and/or partners, often experienced in combination with financial hardship, societal expectations and/or pubertal changes.^8^ Common mental disorders may negatively affect the functioning of adolescent mothers and increase the risk of mental health and behavioural problems in their children.^10^ Furthermore, a study of adolescents from a low-income setting showed that nearly half of pregnant teenagers with positive depression screens had repeat pregnancies within two years of their first delivery, showing a 40% greater risk of subsequent pregnancy than those with no depressive symptoms.^11^ Recurrent pregnancies further place them at risk for adverse physical and mental health outcomes. A review of effective mental health interventions for youth indicates that adolescents have low levels of engagement with and retention in mental health services.^12^ Identifying at-risk youth, diagnosing mental health illness and enhancing utilisation of relevant services among adolescents remain challenging.^13^

The World Health Organization has indicated that there is a strong commitment to preventing early pregnancy and poor reproductive outcomes in adolescents^14^ and has provided guidelines for adolescent-friendly services.^15^ However, there are a limited number of studies published in the area of adolescent utilisation of maternal health services from LMICs.^7^ In these settings, further research is critically needed to understand and account for the experience of adolescent motherhood so that evidence-based interventions may be developed to improve the health of adolescent mothers and their children.^16^

This article contributes to understanding the experiences of adolescents in a low-resource setting who engaged with a mental health service, integrated into maternity care, during and after pregnancy. We sought to explore with pregnant and postnatal adolescents using an integrated mental health service, the barriers and facilitators they experienced in relation to service uptake as well as their preferences for the form the service should take.

## Research methods and design

This was an exploratory-descriptive qualitative study that used semi-structured interviews to explore perinatal adolescents’ perceptions of a mental health service.

### Setting

This study was conducted at three midwife obstetric units (MOUs) in Cape Town, South Africa. At the time of data collection, the Perinatal Mental Health Project (PMHP) had, for several years, provided a screening and psychosocial service, free of charge, for pregnant women attending three MOUs within the Cape Town Metropole. All three MOUs are situated in or serve women coming from low-income, residential suburbs, with high levels of unemployment, poverty and crime. The service offered by PMHP consisted of screening pregnant women attending their first antenatal visit, for symptoms of CMDs and for risk factors for CMD. The screening questionnaires were available in the local languages spoken in the area. PMHP screeners were trained to normalise the screening process by providing an introductory explanation which included rationale for the screening. Women who screened positive for either CMD symptoms or risk factors were referred to a qualified, on-site, mental health counsellor for psychosocial counselling and case management. The intervention consisted of an unlimited number of counselling sessions based on need and one telephonic follow-up session 6–12 weeks after birth. All information related to the women who received the intervention was recorded in an electronic database. A full description of the service, with outcome data, is published elsewhere.^17,18^

### Recruitment

A purposive sampling approach was used. A PMHP researcher, Z.A., mined the PMHP database to identify potential participants. Eligibility criteria included women who (1) were pregnant and aged 15–19 years at the time of mental health screening, (2) had screened positive for a CMD, (3) had received one or more counselling sessions during the past year and (4) had concluded their counselling relationship. A data-extraction form was used to capture demographic and contact information of potential participants, as well as information related to their gestation at screening and the screening and counselling intervention they had received. Sixty-one adolescent women were identified from the database, and numerous attempts were made to contact them telephonically. Forty of the telephone numbers were no longer working or belonged to someone other than the potential participant.

**FIGURE 1:**
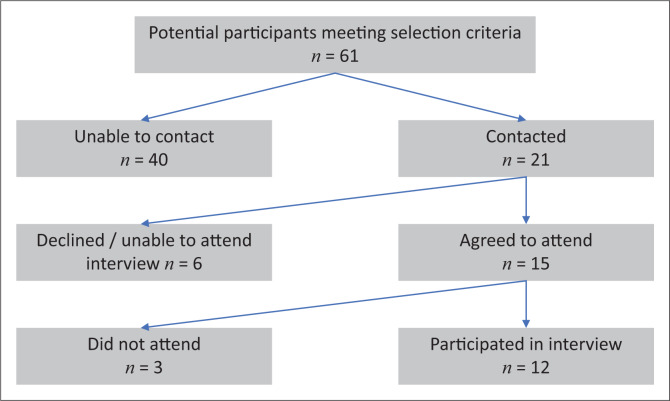
Participant selection flow chart.

Z.A. was able to contact 21 women, of which one refused to participate after this study had been described to her. An additional five women had moved away or were unable to attend an interview because of working hours, and three women who had agreed to participate did not attend the scheduled interview. All participants were either English speaking or fluent in English as a second language and agreed to conduct the interview in English. The participant selection flow is depicted in Figure 1.

### Data collection

A semi-structured, English-language interview guide was developed by Z.A. and reviewed by all authors. The interview questions were aimed at eliciting information on the participants’ experiences of the screening and counselling processes, their perceived barriers and enablers to accessing the mental health service, and the type of mental health services they would prefer. A 23-year-old woman, first-language English-speaking research assistant with a bachelor’s degree in psychology, and who was registered for a master’s degree in medical anthropology, was trained to administer the semi-structured interviews. This person was not employed by the PMHP and had not previously engaged with the participants in any way. She explained to participants that the rationale of the interview was to learn from younger mothers about their experiences of using the mental health service and their thoughts and ideas regarding optimum service design. The interview process took between 45 and 60 min to complete and was audio-recorded. All interviews were conducted in a private space at the MOU where the participants had received antenatal care, with no others present, to ensure confidentiality. Between May 2018 and June 2018, 12 women participated in a semi-structured, qualitative interview. Participants were asked to describe what they could recall of their experience of screening and counselling through the PMHP service, and questions were asked to probe how they felt about and what they understood from their experiences. In assessing barriers and facilitators to accessing mental health services, probes included questions on confidentiality and on emotional and physical factors. The participants were asked to provide their opinion of what kind of help or support teenagers would appreciate when they were feeling sad, worried and alone. They were asked about who would be best placed to provide this support and what form the support should take.

### Data analysis

The semi-structured interviews were transcribed by a first-language English-speaking research assistant who had been trained by the PMHP researcher, Z.A. A framework analysis was used for the data analysis. The development of initial codes was guided by the semi-structured interview topics and followed the framework analysis approach.^19^ Further themes not captured by the initial coding were identified through extensive reading of the transcripts. Transcripts and data were managed using NVivo 12 Pro qualitative data analysis software (QSR International Pty Ltd., Melbourne, Australia). Two researchers, Z.A. and S.F. (with a PhD and master’s degree, respectively), separately analysed the transcripts. Thereafter, the analyses were compared, and a discussion was held to resolve any differences. The researchers agreed that the data were saturated and that further interviews were not required. The credibility of the data was supported by analyst triangulation as well as triangulation of sources; participants were recruited from more than one setting. Confirmability was established by documenting the procedures for checking the data. These techniques supported the trustworthiness of this study.

### Ethical consideration

Ethical approval for the study was obtained from the Human Research and Ethics Committee at the University of Cape Town (HREC REF: 414/2012). The Western Cape Provincial Department of Health approved the use of the research sites (REF No: WC_201708_013). All participants were informed that they were free to withdraw from the study at any time without consequences. Those who participated in the study provided written informed consent after the procedure had been verbally explained to them. Data were de-identified, and interview audio files, transcripts and data files were stored on a password-protected server to ensure data protection. Participants who were identified by trained and supervised researchers to require mental health support were referred to a qualified, on-site counsellor for free services.

## Results

Twelve adolescent mothers were interviewed for this study. The participants’ demographic information is summarised in Table 1.

**TABLE 1:** Demographic characteristics of participants.

Demographic characteristic	Number of participants
**Age**	
15–17 years	5
18–19 years	7
**Marital status**	
Unmarried	10
Married	2
**Home environment**	
Living alone	1
Living with parents or family	7
Living with partner or partner’s family	3
**Occupation**	
High school student	3
Tertiary education student	3
Unemployed	4
Employed	2
**Number of counselling sessions attended**	
1–2	4
3–4	4
5–8	4
**Facility**	
1	7
2	4
3	1

Several themes emerged from analysis of the transcripts. These have been grouped under three broad topics: experience of mental health service use, perceived barriers and facilitators to mental health service use and preferred mental health service design.

## Experience of using the mental health service

The subthemes that emerged under the experience of the mental health service included mental health literacy, screening, resistance to referral, expectations and experiences of counselling.

### Mental health literacy

The participants revealed differing levels of insight around mental health issues. Some participants were able to articulate how they had felt and named the emotion as depression: ‘I think I was kind of depressed … I think I cried for about a month’ (Participant 10, age 19, postnatal, 5 counselling sessions). In explaining how her feelings of depression were manifested, another participant expressed the following:

‘I won’t feel like talking anymore. I’m not the same like the time I was before, and I’m not feeling like talking anymore to people. So that’s why I don’t talk and I don’t walk.’ (Participant 7, age 17, postnatal, 2 counselling sessions)

However, one participant indicated her confusion around diagnostic terminology:

‘She [the screener] was talking about emotions so I was not quite sure what, like anxiety, do you have anxiety? Then I was not even sure what is it. I hear people saying anxiety at school, but I don’t know what it really means.’ (Participant 1, age 18, postnatal, 6 counselling sessions)

When probed, many of the participants indicated that mental health problems were common in their community and they were able to identify those who suffered from mental distress: ‘but you can see … like a lot of people is mentally messed up in this place’, and ‘… they just look lost. Like they living life not really wanting to live’ (Participant 3, age 18, pregnant, 1 counselling session).

### Experiences of mental health screening

When asked about their experience of mental health screening, most participants responded that they had understood the questions and the need for mental health screening. ‘They [*screening questions*] helped me a lot … I think it’s to make sure, for you to be healthy, because also stress can affect your baby’ (Participant 1, age 18, postnatal, 6 counselling sessions).

Only one participant felt that the questions were too personal, which made her uncomfortable. Because of the routine nature of the screening, she felt that she had no choice but to answer. Several participants reported that the routine screening normalised the context and made the questions about mental health issues more acceptable:

‘… There was those routines you have to go through before you give birth and they called me … and it was more of a background check. Like to see how things worked out when I found out that I was pregnant.’ (Participant 1, age 18, postnatal, 6 counselling sessions)

The attitude of the screener played an important role in participants’ perceptions of the acceptability of screening:

‘… She made you feel comfortable. It was like, I could tell her anything. So, the answers I gave her was just honest answers … It was quite simple. It wasn’t like that in too deep. Like she didn’t pry too much into your personal stories. She kept it on a normal level.’ (Participant 3, age 18, pregnant, 1 counselling session)

However, for others, the mental health screening was the first time that they had comprehended the implications of being pregnant, and it was an emotionally charged experience:

‘… It was a very emotional process. It was when I realized I wasn’t happy with my pregnancy and I wasn’t happy with a lot of things, so it was sad.’ (Participant 10, age 19, postnatal, 5 counselling sessions)

### Resistance to referral

In this study, several participants expressed initial resistance to being referred for counselling:

‘… She asked me about, um, will I like to go for counselling, so I tell her no, so she asked me why, so I tell her I’m not feeling so, and she was also asking me do I need help …’ (Participant 7, age 17, postnatal, 2 counselling sessions)

and:

‘I am that person that doesn’t like to talk to anyone. Like, I keep quiet and go through these things on my own.’ (Participant 11, age 19, postnatal, 5 counselling sessions)

### Expectations about counselling

No participants had previous experience of therapeutic counselling. Most of the participants were worried about engaging with the counsellor and fearful of counselling, expecting to be judged or be bored by probing into their private lives. One participant feared that she could have her baby taken away:

‘… I thought they were going to tell me about how young I am. I thought they judge a person or something, that was why I did not want to talk when I saw her the first time.’ (Participant 5, age 15, postnatal, 3 counselling sessions)

and:

‘… Expecting it to be different. For me it was like whatever, I’m just gonna go, yeah, whatever they tell me I’m just gonna say ‘mmm yea’ to everything, I’m gonna talk like that. And then when I went to see her it was different.’ (Participant 2, age 17, postnatal, 8 counselling sessions)

### Experience of counselling

Once engaged with the counselling process, all participants reported positive experiences and valued being listened to, understood, being able to discuss their problems freely and feeling validated. This was expressed by some as having someone ‘*on your side*’:

‘… I was free to talk about my problems. She never gave me difficulties when she was asking the questions. She would put herself in my shoes like trying to ask me the questions in a way that um, that would not hurt me or something like that.’ (Participant 11, age 19, postnatal, 5 counselling sessions)

Positive outcomes were also reported. A participant expressed that the experience of counselling had enabled her to speak more openly with her parents. Another indicated that she felt ‘closure with my past’ and a further participant indicated that it had helped her ‘to process things’. Many participants commented on the skills they had learnt and how they had new coping mechanisms. ‘… I’m learning. I’ve improved so much, even sometimes when I feel upset, I just remember ok [counsellor] taught me this or taught me that’. (Participant 1, age 18, postnatal, 6 counselling sessions) While we probed to investigate whether demographic factors of the counsellor were relevant for the participants in any way, these did not appear to be so.

## Perceived barriers and enablers to accessing mental health services

The subthemes related to accessing mental health services pertained to either or both barrier factors and enabling factors. They included stigma, confidentiality, access, coordinated appointment times, disclosure of attending counselling and social support.

### Stigma

Stigma was raised as a significant barrier to accessing services. The participants felt that they would be judged for being young and pregnant and mocked for having mental health problems. They indicated that there was significant stigma around both teenage pregnancy and mental health issues in their communities.

Regarding pregnancy, the following comments reflect their perceptions:

‘… When you get pregnant at a young age, most people are going to judge you.’ (Participant 11, age 19, postnatal, 5 counselling sessions)

and

‘… They would judge you, like no, it was bad of you to do this, um, it was irresponsible. If you’re being pregnant, then why are you suffering, you see, you should have gotten pregnant when you were ready.’ (Participant 9, age 19, postnatal, 2 counselling sessions)

The fear of being talked about and being judged for admitting to having a problem was raised by almost all participants:

‘… If I tell you, you will go and tell your friend and your friend will tell a friend, so maybe you will walk and they will be pointing fingers at you, you know? So that’s something that I don’t really show my emotions.’ (Participant 1, age 18, postnatal, 6 counselling sessions)

and:

‘… First of all, friend’s judge, they judge and when you tell them something, they go tell their mothers. You see, once you tell people you are seeing a counsellor, they assume that you are having big problems, so I didn’t want them to assume that about me.’ (Participant 10, age 19, postnatal, 5 counselling sessions)

### Confidentiality

Participants of our study indicated that they were initially very wary of speaking openly and that the counsellor reassured them that their conversation would remain private. Furthermore, one participant indicated that as the counsellor was not from her community, and this provided further reassurance of confidentiality:

‘… I know what I say will stay here, it will not go. It was confidential. I was so happy because she didn’t know me, she didn’t know any friend of mine or family member that she could go and gossip to me about.’ (Participant 10, age 19, postnatal, 5 counselling sessions)

### Physical access

In this study, several logistical and environmental factors were reported as barriers to service access in general and not particular to uptake of mental versus physical health services. These included the health facility being situated in a dangerous environment, ‘the first time I was coming here, we got robbed. I was scared to come’, (Participant 4, age 19, postnatal, 6 counselling sessions) not having money for transport or having to take several taxis in order to reach the facility.

### Coordinated appointment times

The PMHP service attempted to coordinate the timing of counselling appointments with regular antenatal check-ups. This integration of appointment times was noted as a facilitator to accessing mental healthcare. It ensured that young women with few resources did not have to spend extra resources to access both services. ‘… Because then you don’t need to go out of your way to go for the counselling. Because it is on your [*antenatal*] appointment day’ (Participant 3, age 18, pregnant, 1 counselling session).

### Disclosure of attending counselling

During counselling, all the participants were encouraged to disclose that they were receiving counselling to a supportive adult. All participants indicated that they had disclosed to an adult family member that they were attending counselling. This elicited emotional support for some and practical support for others, such as reminders about appointment times or support with transport to attend appointments.

### Social support

Social supports, from family and from school, were raised as being important facilitators to help-seeking. With regard to school, one participant reported: ‘It was very difficult, but my teachers never gave up on me. They kept encouraging me to never give up. They kept telling me they know my potential’ (Participant 10, age 19, postnatal, 5 counselling sessions). Another participant reflected, ‘I told myself that if my mother has accepted me and she is giving me all the support she can, I don’t care who says what’ (Participant 12, age 17, postnatal, 3 counselling sessions).

## Preferred mental health service design

All participants mentioned that individual face-to-face counselling would be their preferred format for a mental health intervention. They explained that the attitude and engagement of the counsellor were crucial to this experience. As indicated by one of the interviewees: ‘You must make her feel like she’s not alone, like there is people out there that care’ (Participant 3, age 18, pregnant, 1 counselling session). The counsellors were perceived as being ‘warm’, ‘sweet’, ‘a quiet person who will just listen to you’, ‘I feel like she knows me’, ‘very welcoming’, ‘she does not judge’, ‘she approaches you with respect’ and ‘I felt like she cared.’

Some of the participants supported the idea of a peer group – a forum where young pregnant women could share their experiences. However, other participants argued that they would feel uncomfortable in a group setting and that trust would be problematic for them.

## Discussion

The findings indicate that adolescents perceive considerable stigma around both teenage pregnancy and mental illness, inhibiting their use of mental health services. Other barriers to service uptake include the fear of a lack of confidentiality as well as logistical and environmental obstacles. Service uptake was facilitated when adolescents had support from trusted adults in their lives and when appointment times were flexible. Face-to-face individual counselling was their preferred format for a mental health intervention. Positive experiences of counselling were linked to non-judgemental attitudes and supportive engagement from counsellors.

Mental health literacy refers to an individual’s knowledge and beliefs about mental conditions which may assist in identification, management or prevention. It also refers to knowledge and strategies to support and manage oneself or others experiencing mental health conditions.^20^ The participants in this study showed varying levels of mental health literacy with some participants being able to name their emotion or describe how their feelings were manifested. Similar expressions of withdrawal and descriptions of depression were expressed in a study of adult pregnant women from an urban, low-income South African setting, similar to ours.^21^ Low levels of mental health literacy have been identified in adult perinatal women attending antenatal care in an impoverished, urban South African area.^22^ Future research may be able to assess whether there are any educational or socio-economic predictors of mental health literacy.

While some participants were not easily able to articulate their understanding of mental illness, they were aware of and able to identify mental distress in others. In a systematic review, Gulliver et al. indicated that poor mental health literacy and difficulties in recognising symptoms are barriers to adolescents accessing mental health services.^23^ Thus, a crucial step in improving access to mental healthcare for adolescents would be to raise awareness of mental health through multiple channels such as the health and education systems, and through social and traditional media platforms. Intersectoral collaboration has been highlighted as a facilitator to improving adolescent mental health service use in LMICs.^24^

Routine mental health screening was both understood and valued by our participants. As the screening questionnaires were available in the local languages, this might have contributed to their understanding of the screening questions. While there is debate around routine screening for CMDs in the perinatal period, the arguments for screening include early identification and referral for treatment.^25^ Furthermore, there is strong indication of acceptability of mental health screening in similar adult populations in South Africa^26,27^ and in systematic reviews of psychosocial assessments for young people.^28^ Initial interactions between care providers and adolescents provide a platform for all future engagement with mental health services, and eventual outcomes may be dependent on the processes occurring at this point.^29^ This highlights the importance of the screeners’ attitude and method of engagement with adolescents in determining the likelihood of future mental health service uptake.

Resistance to seeking mental healthcare is not limited to adolescents, but proportionately fewer young people seek help.^23^ In this study, several participants expressed initial resistance to being referred for counselling. This sentiment concurs with research from a multi-country study on adolescent health service use, where the researchers reported that more than 30% of adolescents in Johannesburg, South Africa, did not seek any form of healthcare (incorporating mental and physical healthcare) even when they were aware it was needed. Qualitative data from the Johannesburg study site indicated that adolescents exhibited a general lack of trust in healthcare providers across all service platforms, citing care providers as rude and judgemental.^30^ A lack of knowledge about mental health services has been shown as a barrier to adolescent help-seeking behaviour.^23^ This was demonstrated by participants in our study when they described their expectations of counselling.

Fear about the counselling process was expressed by many of the participants. In a systematic review of 22 studies, the fear of the unknown and the fear of being judged were reported by adolescents as a barrier to mental health service use, while a previous positive experience of mental health services was shown to be a facilitator to service uptake.^23^ This concurs with our evidence and demonstrates the importance of quality engagement between the adolescent and the person providing the referral to mental health services. The referrer needs to provide an explanation of the services offered, the rationale for the referral, and explore with the adolescent any fears or expectations she may have regarding attending mental health services.

Once engaged with the counselling process, all participants reported positive experiences and valued being listened to, understood, being able to discuss their problems freely and feeling validated. The attributes of the counsellor played a significant role in how the participants viewed the counselling process. A non-judgemental and open attitude and the ability to listen, understand and relate to the problems discussed were important to facilitate engagement with counselling. This is supported by other research that indicates that the characteristics of the provider (which include race, credibility, whether they are known or unknown) as well as attitudes (such as confidentiality and judgement) play a deterring or enabling role in service uptake for adolescents.^23^ While we probed to investigate whether demographic factors of the counsellor were relevant for the participants in any way, these did not appear to be so.

Stigma was raised as a significant barrier to accessing services. In many settings, various societal, cultural and religious factors create a prohibitive environment with embedded disapproval of adolescent sexual activity, often demonstrated through stigmatisation and judgemental attitudes towards sexually active adolescents.^31^ In interviews with young mothers in another South African study, Mjawara et al. revealed that young mothers felt ostracised by the wider community.^32^

Mental illness confers a separate and additional stigma. Adolescent women have reported perceiving extreme stigma around psychiatric illness, particularly depression.^33^ A review on adolescent mental health service use indicated that stigma and embarrassment about help-seeking emerged, from both qualitative and quantitative studies, as the greatest barrier to seeking help for mental health conditions.^23^ Addressing stigma around both mental health and adolescent pregnancy is a significant challenge. Service provider attitudes play an important role: negative staff attitudes discourage help-seeking and reinforce stigma.^34^ In South Africa, the abuse of patients in maternity settings has been raised as a significant concern^35^ with adolescents particularly being at risk of victimisation.^36^ Providing adolescent-friendly services would need to include sensitisation of care providers to the needs of adolescents and challenge stigma around sexual activity and mental illness.

Confidentiality and trust in the counsellor were raised as major concerns by the participants and have been raised in other studies as significant barriers or enablers to help-seeking behaviour for adolescents.^23^ In a multi-country study of adolescent help-seeking behaviour in low-income settings, across all sites, adolescents reported that the lack of trust in healthcare providers and in other adults in the community was a key factor that prevented them from seeking care.^30^ While confidentiality is key to any therapeutic relationship, special attention needs to be paid to reassure adolescents and create an atmosphere of trust.

In this study, several logistical and environmental factors were reported as barriers to service access in general, and not particular to uptake of mental versus physical health services. These findings, regarding lack of access, have similarly been reported in other studies for adults in Southern Africa seeking human immunodeficiency virus (HIV) care^37^ and with adolescents in general health-seeking.^30^

The integration of appointment times for counselling and antenatal care was noted as a facilitator to accessing mental healthcare. Other research has indicated that teenaged parents may be more likely to engage in mental health treatment when appointments are flexible and accommodating to their school schedules and allows them to discuss their problems at their own pace.^10^

Support, either emotional or practical, from a trusted adult enabled greater access to the mental health service for the participants. This aligns with other findings that adult support from the home increases adolescents’ likelihood to seek healthcare.^30^

Social supports from family and from school were raised as being important facilitators to help-seeking. While facilitators to help-seeking are comparatively under-researched, there is evidence that young people perceived positive past experiences with services, and social support and encouragement from others as aids to the help-seeking process.^23^

All participants indicated that individual face-to-face counselling would be their preferred format for a mental health intervention. This may have been because of their otherwise limited exposure to other options, and to the positive experience they experienced through the PMHP service. They indicated that the attitude and engagement of the counsellor were crucial to this experience. Other studies have indicated that the healthcare providers need to be trained to adopt non-judgemental attitudes, maintain confidentiality and engage with adolescents, while maintaining communication with families.^3^ Furthermore, a South African study researching health service factors that influenced adolescent retention in HIV care showed that staff-related factors – staff who were kind and who had time for adolescents – increased the odds for retention in services by 2.5 times.^38^

Some of our participants supported the idea of a peer group, a forum where young pregnant women could share their experiences. Other research reported that adolescent mothers appreciated being part of a peer group,^39,40^ and a review indicated that group interpersonal therapy (IPT) has shown success in reducing rates of depression in perinatal adolescents in low-income settings.^41^ However, other interviewees argued that they would feel uncomfortable in a group setting and that trust would be problematic for them. These sentiments are reflected in research by Bledsoe et al., who report that there is limited evidence to suggest pregnant adolescents’ preference for group-based services. Their complicated lives often interfere with attending group interventions, and the experience of trauma, common in this group, is counterindicative of group interventions.^33^

Interestingly, none of the participants mentioned information or communication technology as a potential aid to service design. As the study sites were located within low-resource settings, this may be attributed to their limited exposure to such interventions. However, there is promising evidence from global literature for the benefits of on-line media, where text-messaging has been used to link patients with clinicians, internet-based education has been used to reduce stigma and social media used for youth attitude changes.^12^ There is also evidence that supports adolescent-friendly, internet-based prevention and treatment programmes for depression and anxiety.^42^

## Limitations

This study is limited to adolescent participants who engaged with the maternal mental health services provided by the PMHP in low-resource settings, and thus, the findings may not be generalisable to other populations or adolescents who have experience of other mental health services. We did not interview adolescents who had declined referral to counselling or those who had engaged with obstetric services at other sites. Their views may have provided additional insight into the barriers and facilitators to accessing mental health services. Furthermore, the researchers involved in the analysis were employed by the PMHP and may thus have a biased and favourable interpretation of the data. No external audit of the research was conducted, limiting the dependability. Because of logistical constraints, the transcripts were neither returned to participants for comment or correction, nor were they consulted on the findings of the analysis.

## Conclusion

This article adds an understanding of the experience of adolescents in low-resource settings who engaged with a mental health service during and after pregnancy, the barriers and facilitators to mental health service access as well as the type of services preferred by this group. Our findings indicate that the key components for adolescent-friendly mental health services would be integration of routine mental health screening into existing obstetric services to de-stigmatise mental health problems and optimise screening coverage; coordination of obstetric and counselling appointment times to maximise access to counselling and rationalise the use of limited resources; sensitisation of care providers to the needs of adolescents to reduce stigma around adolescent sexual activity and mental illness; recruitment and training of counsellors who are aware of the particular vulnerabilities and difficulties faced by pregnant adolescents. A non-judgemental, caring and confidential relationship between counsellors and clients is crucial for successful interactions.
